# Geographical Variation in Lip Print Patterns Among Adolescents from the High Andean Quechua and Suni Regions in Perú

**DOI:** 10.1016/j.identj.2026.109416

**Published:** 2026-02-05

**Authors:** Juan Espinoza, Cesar Mauricio-Vilchez, Julia Medina, Fran Espinoza-Carhuancho, Ivan Calderon, Arnaldo Munive-Degregori, Frank Mayta-Tovalino

**Affiliations:** a Faculty of Dentistry, Research, Innovation and Entrepreneurship Unit, Universidad Nacional Federico Villarreal, Lima, Perú; b Bibliometrics Evidence Evaluation and Systematic Reviews Group (BEERS) Human Medicine Career, Universidad Científica del Sur, Lima, Perú; c EVIDENTIA Research Group, Faculty of Dentistry, Universidad Nacional Mayor de San Marcos, Lima, Perú; d Vicerrectorado de Investigación, Universidad San Ignacio de Loyola, Lima, Perú

**Keywords:** Adolescents, Lip prints, High Andean, Sex differentiation

## Abstract

**Objective:**

The aim of this study was to determine the lip print patterns among adolescents from the Quechua and Suni ecological regions of Ancash, Peru, and to explore the relationship between the variables and their differentiation using logistic regression models.

**Methods:**

A quantitative, observational, prospective, cross-sectional, and analytical study was conducted. The sample comprised 192 adolescents from representative educational institutions, who requested the informed consent of their relatives. Personal and anthropometric data were recorded, and lip prints were obtained according to the conventional system of lipstick and adhesive tape using the classifications by Suzuki &Tsuchihashi and Renaud.

**Results:**

The population was predominantly male (55.2%) and more represented from the Quechua population (61.5%). Regarding their nutritional status, it was found that more than half were in average nutritional status (53.6%), although a significant proportion was overweight (41.8%). Regarding the Suzuki patterns, there was a predominance of types II (52.1–55.8%) and IV (50.5%), while the Renaud patterns presented a predominance of types G (49% in the upper left quadrant) and E (36.4–38.1%) in the lower quadrants. In the multivariate analysis, it could be identified that the only factor significantly associated with the Suzuki pattern was the geographical region. Adolescents from the Suni region presented this pattern more frequently than those of the Quechua population (60% lower odds, compared with Quechua adolescents: OR = 0.40; 95% CI: 0.22–0.73; p = 0.003). Sex, age, weight and height had no significant associations.

**Conclusion:**

The results indicate that lip print patterns have a characteristic dominant pattern in high Andean adolescents, with a significant influence of the geographic region on the morphology of the lips. The results underscore the significance of lip prints as a biometric method and offer proof of the morphological diversity of the Andean populations.

## Introduction

The identification of individuals is a crucial component of forensic science, as it allows for the clarification of criminal acts, the management of emergencies in mass disasters, and the resolution of cases where establishing an individual’s identity is essential.^1^ Thus, in the forensic field, identifying individuals mean contributing significantly to reducing the number of possible matches between individuals. To achieve these identification results, science has promoted the application of traditionally reliable methods over the years, including fingerprinting, genetic analysis, and forensic dentistry, among others.^2^

In recent years, according to Loganadan et al,^3^ dentistry has sought to use the study of lip prints as a complementary identification technique that is highly accurate, since the presence of groove patterns on the lips has unique and permanent characteristics in each person, similar to fingerprints. In this sense, cheiloscopy is a great contribution of dentistry as a forensic method.

Sex determination is a fundamental aspect of forensic investigation, and lip print analysis has become a potential tool. Certain studies using the Suzuki and Tsuchihashi classification system demonstrated specific variations in lip print patterns according to sex, with some patterns being more prevalent in men and others in women.^4^ Likewise, Mishra et al^5^ indicated that it is not only possible to note variations in gender but also distinctions in the distribution of lip patterns between different geographical regions, which underscores the importance of considering population origin in forensic studies of this nature.

Along these lines, some studies^6^ indicate that in different high-altitude geographical regions, as well as in Andean regions, where environmental conditions include variations in atmospheric pressure and, therefore, a decrease in oxygen, morphological adaptations may occur. For example, in a geographical context like that of Perú, Cabezas et al^7^ have shown that indigenous Ecuadorian children living in high-altitude areas have actinic skin lesions on their lips. These alterations could modify the configuration and distribution of the lip grooves, affecting the effectiveness of the traditional lip print classification methods. This justifies the need for specific forensic studies in the Andean geographical areas.

In addition, the methodology used in the collection of lip prints has a direct influence on the quality of the characteristics that are obtained. Classical techniques with scientific chemical and physical methods and the modern digital techniques can yield different results if they are not applied uniformly. In this sense, the quality of the latent conjunctive of the fingerprints can differ in view of the system used. Because of this, the necessity of using uniform systems that should be validated by the forensic sciences to ensure reliability in the analysis of the lip prints according to the geographic localization becomes obvious.^8^

Thus, in this order, it is important to establish the sex differentiation by means of the lip prints patterns of the adolescents grouped in the Quechua and Suni natural areas of Ancash, with the application of the classification systems of Suzuki & Tsuchihashi and Renaud.

## Materials and methods

### Ethics

This research was conducted in strict compliance with the ethical principles established by the Federico Villarreal National University, previously approved by the Ethics Committee, which issued Resolution No. 079-03-2025. Since this study worked with adolescents, so that there was strict adherence to the principles of the Helsinki Declaration, it was ensured that the protection of the rights, dignity and well-being of the study subjects was guaranteed.

### Type of research

This is a quantitative, observational, prospective, cross-sectional and analytical study. To ensure transparency and methodological quality, the results were reported according to the recommendations of the Strengthening the Reporting of Observational Studies in Epidemiology (STROBE) guidelines.^9^

### Sample size and temporal and spatial scope

The available adolescent students of both educational institutions were included, giving *N* = 192 participants. The investigation was carried out from January to October 2025 in order that there should be enough time to include and to study the target population. The investigation was conducted in two secondary educational institutions of the province of Huari of the department of Ancash, Perú. The first was the Educational Institution César Vallejo, which is in the district of Huántar and pertaining to the Quechua natural region, and the second was the Educational Institution Nuestra Señora de Guadalupe located in the town of Huamparán corresponding to the Suni natural region. The selection of these sites enabled us to compare the patterns in two different geographical and cultural environments within the same province, thus lending diversity and representativity to the study sample.

### Selection criteria

To form the sample, the inclusion criteria considered were students enrolled in the selected educational institutions, from the Quechua and Suni natural regions, who also had the informed consent signed by their parents or guardians. Participants with visible injuries, scars, or pathologies on their lips that would make it difficult to obtain an adequate lip print were excluded, as were incomplete records in the data collection forms and adolescents who did not have the informed consent of their parents or guardians.

### Instruments

Data collection consisted of a direct observation of the lip print patterns, recorded on forms designed expressly for this study, showing both the prints and personal data of the people taking part in this experiment. These orders will provide the information on all of the prints so that they can be arranged and classified further ap- The prints will be arranged and classified by the Suzuki and Tsuchihashi systems by a necessary quadrant analysis in the following manner, ie, upper right, upper left, lower right, lower left, this terminology is well and wisely gone over in the various forensic studies and also there are contemporary studies done on modern methods.

### Procedures

**Fig. 1:**
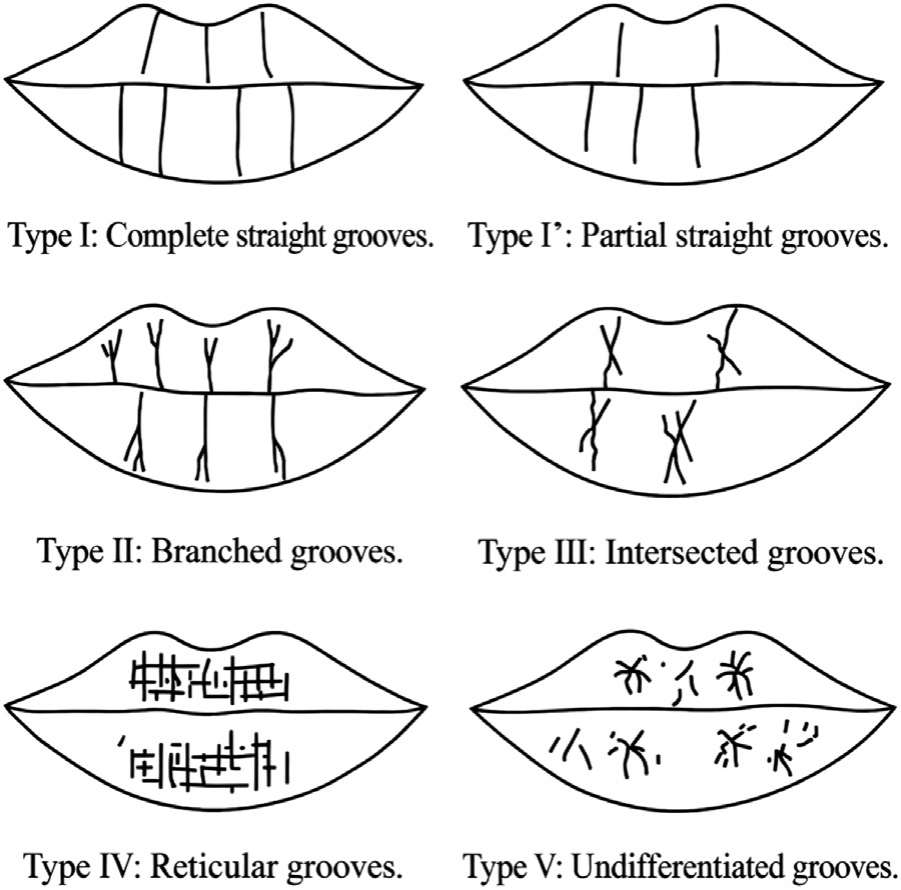
Types of lip print patterns according to the Suzuki and Tsuchihashi classification. Classification of lip prints according to their morphology (X-X’, the midline. Y-Y’, the horizontal line), (Venkatesh and David^17^).

**Fig. 2:**
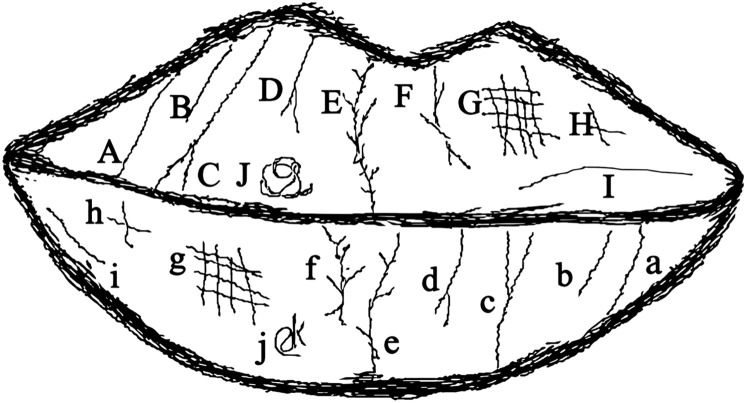
Types of lip print patterns according to the Renaud classification. The current classification model considers ten different lip print patterns (Ata-Ali and Ata-Ali^18^).

To strengthen the methodological rigor of the lip print classification, we wanted to include intra-observer reliability measures to ensure consistent agreement amongst raters. We then used a blind classification layout to continue to reduce the potential for bias from prior exposure to the participants’ characteristic features. The rationale for employing both the Suzuki & Tsuchihashi system and the Renaud system simultaneously stems from their philosophical divergence with respect to how they describe lip prints. While the Suzuki & Tsuchihashi systems focus on identifying the principal patterns within lip prints, the Renaud System identifies the subtle details of wrinkle configurations and adds richness to the morphological interpretation while reinforcing the robustness of the findings (Fig. 1, Fig. 2).

To ensure accuracy and biosecurity, anthropometric and forensic recording instruments were used. The weight was obtained from electronic scales and the height from a height meter. The lip prints were obtained with transparent adhesive tape with fluorescent lipsticks to visualize the grooves and the pits. Sterile swabs for single use were provided to each participant, the evaluator with gloves and a mask, to guarantee hygienic and protective conditions during the study.

Before executing the study, institutional permissions were obtained from letters of introduction signed by the Faculty of Dentistry of the National University of Federico Villarreal, addressed to the authorities of the participating educational institutions. Subsequently, informed consent was given to the parents or guardians to explain the objectives and range of the research.

The personal data of the students were collected (name, age, sex and origin) from the studies school records, as well as from anthropometric measurements of weight and height. The lip prints were taken, for which the lips were cleaned and prepared before putting on the lipstick and the print was taken with adhesive tape, with each record being kept in laminated sheets. At the end of the work, a humid cloth was given to the participants to eliminate the lipstick that was called to have on their lips.

Finally, the obtained prints were classified directly in the observation forms, coded according to the reference systems and systematized in a databank that guarantees the correlation between each print and its participant, guaranteeing the quality and precision necessary for the posterior statistical analysis.

### Analysis plan

The database was imported to the statistical program Stata 17.0, where the consistency of the database and the encoding of all the variables was checked. Categorical variables (sex, natural region, nutritional status, and lip print patterns) appeared in exclusive categories and the continuous variables (age, weight, height, etc.) appeared in a ratio scale that enabled their evaluation in the income of each analysis. The plan involved a descriptive contribution of absolute frequencies and percentages and an inferential analysis with logistic regression, in which the dependent variables of the lip type patterns (simple and complex) were analyzed against the independent variables of sex, age, height, weight, food state, and natural region. The results are shown as odds ratios (OR), confidence intervals of 95% (95% CI), and an index of significance (*P* < .05).

## Results

**Fig. 3:**
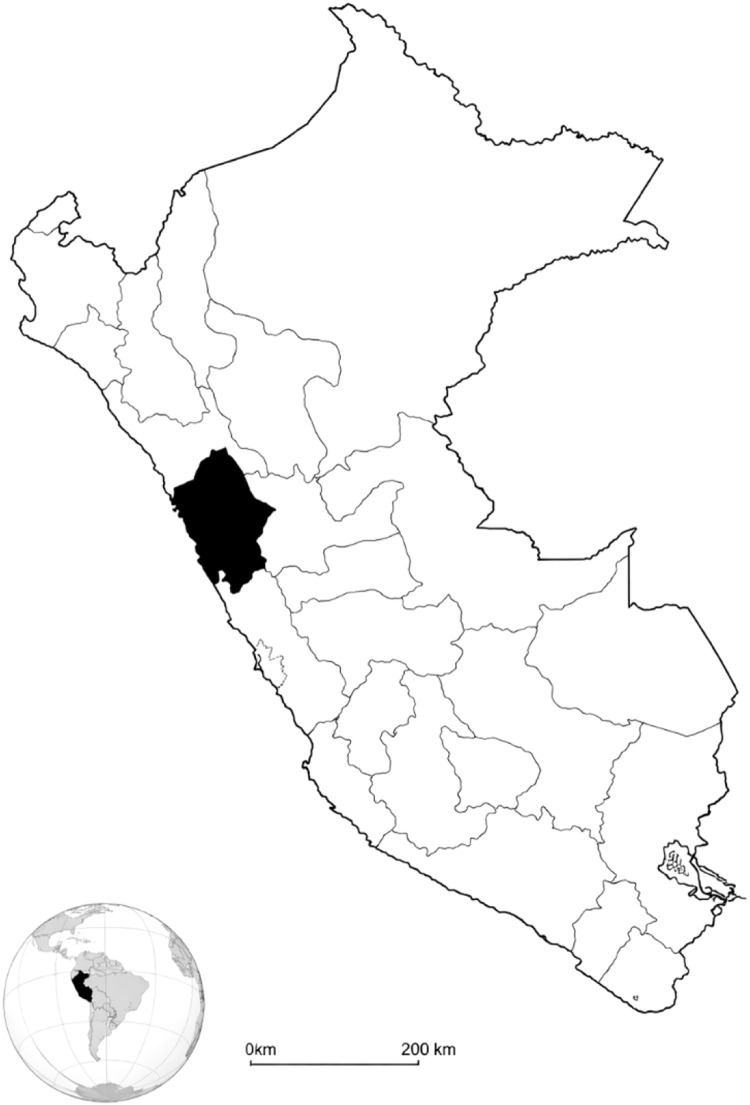
Geographical location of the Department of Ancash in Perú.

The study population was made up of 192 adolescents with more males (55.2%) than females (44.8%). Most belonged to the Quechua region (61.5%) and 38.5% to the Suni region; thus, both high Andean geographical contexts were fully represented. In what corresponds to nutritional status, there was a majority with their weight within normal levels (53.6%), but with a considerable percentage amount of excess weight outside normal levels: 27.7% overweight and 14.1% obese. Cases of malnutrition were infrequent, with 4.1% underweight and only 0.5% severely underweight (Table 1) (Figure 3).

**Table 1:** Distribution of sex, geographic region, and nutritional status in the study population.

Variable	f	%
Sex		
Female	86	44.8%
Male	106	55.2%
Geographic region		
Quechua	118	61.5%
Suni	74	38.5%
Nutritional status		
Severe thinness	1	0.5%
Thinness	8	4.1%
Normal	103	53.6%
Overweight	53	27.7%
Obesity	27	14.1%

An analysis of the lip traces in the subjects, classified according to the Suzuki and Tsuchihashi classification, showed marked differences between the quadrants. The most frequent type was II in all positions, representing more than one-half of the impressions in the lower right quadrant (55.8%) and lower left quadrant (52.1%), substantiating its dominating character in the total of impressions (331). Type IV was also well marked, especially in the upper left quadrant (50.5%), with tightening its grip as the second most prevalent pattern (221). Types I and III had moderate representation but were exceedingly more common in the lower quadrants (24.5% and 7.3%, respectively). Types I' and V were rare, appearing at frequencies of less than 2% in all quadrants, indicating their minor importance in this population. It may be concluded from these observations that types II and IV are the prevalent patterns in adolescents under consideration, while types I, III, I' and V are the less frequent varieties (Table 2).

**Table 2:** Lip print patterns according to the Suzuki & Tsuchihashi classification system.

Suzuki & Tsuchihashi	Upper left	Upper right	Lower left	Lower right	Total
	f (%)	f (%)	f (%)	f (%)	
**I**	32 (16.7)	27 (14.1)	47 (24.5)	42 (21.9)	148
**I’**	1 (0.5)	0 (0.00)	3 (1.6)	3 (1.5)	7
**II**	48 (25)	76 (39.5)	100 (52.1)	107 (55.8)	331
**III**	14 (7.3)	20 (10.4)	14 (7.3)	10 (5.2)	58
**IV**	97 (50.5)	69 (35.9)	27 (14.1)	28 (14.5)	221
**V**	0 (0.0)	0 (0.0)	1 (0.5)	2 (1.1)	3

An analysis of the patterns of wrinkles of the lips according to the classification of Renaud showed a marked predominance of the G and E types in the population studied. The G type was the most frequent in the superior left quadrant (49%) and was found in very considerable proportions in the superior right side (33.4%), giving a total of 210 records. The E type, on the other hand, was predominant in the inferior quadrant, being found in 36.4% of the left side and 38.1% of the right side, giving a total of 201 records, thus graphically demonstrating its predominance around the lower lip. The types A and D also presented a very considerable record, especially in the superior quadrants, giving 145 and 94 records, respectively. The types, on the contrary, of B, I and J were very rare and, in every quadrant, gave frequencies below a limit of 2%, which stands out as remaining in the marginal position in this sample. That is, then, G and E were the more representative configurations of the wrinkles of the lips in the adolescents studied, while the other types appeared in a secondary or residual manner (Table 3).

**Table 3:** Lip print patterns according to the Renaud classification system.

Renaud	Upper left	Upper right	Lower left	Lower right	Total
	f (%)	f (%)	f (%)	f (%)	
**A**	33 (17.1)	29 (15.1)	42 (21.9)	41 (21.3)	145
**B**	1 (0.5)	0 (0)	3 (1.5)	3 (1.5)	7
**C**	10 (5.2)	18 (9.3)	12 (6.2)	8 (4.1)	48
**D**	21 (11)	22 (11.4)	25 (13.1)	26 (13.5)	94
**E**	18 (9.3)	40 (21)	70 (36.4)	73 (38.1)	201
**F**	5 (2.7)	9 (4.6)	7 (3.6)	6 (3.1)	27
**G**	94 (49)	64 (33.4)	27 (14.1)	25 (13.2)	210
**H**	10 (5.2)	10 (5.2)	4 (2.1)	7 (3.6)	31
**I**	0 (0)	0 (0)	2 (1.1)	1 (0.5)	3
**J**	0 (0)	0 (0)	0 (0)	2 (1.1)	2

In the multivariate logistic regression model evaluating factors associated with the identification of the global Suzuki lip-print pattern, only the geographic region demonstrated a statistically significant association. Adolescents from the Suni region had 60% lower odds of presenting the global Suzuki pattern compared with those from the Quechua region (OR = 0.40; 95% CI: 0.22-0.73; *P* = .003). This finding suggests a meaningful population-based or ancestral influence on the lip-print morphology among high-Andean groups. Sex and age were not significantly associated with the pattern (OR = 0.67; *P* = .190 and OR = 1.04; *P* = .701, respectively), indicating that biological sex and variation within the adolescent age range do not substantially contribute to differences in the global Suzuki configuration. Similarly, weight showed no significant association (OR = 1.01; *P* = .659), suggesting that general body mass has no measurable impact on lip-print characteristics (Table 4).

**Table 4:** Factors associated with lip patterns obtained using the Suzuki & Tsuchihashi classification system.

Variable	OR	*P*	95% CI
Ref: Sex			
Male	0.67	.190	0.37-1.22
Age (years)	1.04	.701	0.84-1.30
Ref: Region			
Suni	0.40	.003	0.22-0.73
Weight (kg)	1.01	.659	0.97-1.05

In the multivariate logistic regression model evaluating the factors associated with the Renaud global lip-print pattern, none of the included predictors showed statistically significant associations with the outcome. Sex was not related to the probability of presenting the Renaud pattern (OR = 0.63, *P* = .504), indicating that males and females exhibited comparable distributions. Age demonstrated a trend toward a negative association (OR = 0.63, *P* = .071), although this effect did not reach statistical significance. Likewise, residing in the Suni region was not associated with the pattern compared with adolescents from the Quechua region (OR = 0.91, *P* = .892). Weight also showed no meaningful influence on the likelihood of this lip-print classification (OR = 1.05, *P* = .287) (Table 5).

**Table 5:** Factors associated with lip patterns obtained using the Renaud classification system.

Variable	OR	*P*	95% CI
Ref: Sex			
Male	0.63	.504	0.16-2.45
Age (years)	0.63	.071	0.38-1.04
Ref: Region			
Suni	0.91	.892	0.24-3.42
Weight (kg)	1.05	.287	0.96-1.15

## Discussion

This study analyzed the relationship between lip print patterns and gender in adolescents from the Quechua and Suni regions in the department of Ancash, applying the Suzuki & Tsuchihashi and Renaud systems. At a descriptive level, it was observed that, although both systems consider multiple configurations, the distribution of lip patterns varied according to the classification system. In the Suzuki and Tsuchihashi system classification, patterns II and IV predominated, whereas in the Renaud system classification, patterns G and E were more frequent. This reflects that, despite the existence of several categories, the sample generally tended to group into recurring configurations.

In the logistic regression model, the upper quadrants of the Suzuki and Tsuchihashi system reached significance, as the upper left quadrant increased the probability within the model fivefold and the upper right quadrant almost sixfold. However, the lower quadrants and all those in the Renaud system were not significant. On the other hand, associated factors such as age, geographic region, height, weight, and nutritional status also showed no statistical relevance. The overall pattern summarizing the classification of both classification systems into simple and complex patterns indicates that the Suzuki & Tsuchihashi model presented an adequate fit (Pseudo R² ≈ 0.71), while Renaud’s model was not significant (Pseudo R² ≈ 0.05).

Several studies^1^, ^2^, ^3^, ^4^, ^5^, ^6^, ^7^, ^8^, ^9^, ^10^ have shown that the usefulness of cheiloscopy in sex differentiation depends both on the classification system used and on population characteristics. Meena et al in India found that the upper quadrants had greater discriminatory potential between sexes. These results partially coincide with the present study, as the upper quadrants also acquired statistical significance but within the overall pattern model.

Similarly, Alqarni et al^10^ Argued that sex differences tend to be localized and not generalized throughout the lip region, a finding that coincides with the limitation observed in our sample. Likewise, Thermadam et al,^11^ in a large sample of more than 2112 individuals, highlighted the variability of the quadrants between different populations, thus evidencing the discrepancies between studies from different regions.

In fact, Vanguru et al^12^ showed the hereditary correlations between patterns of palm and lip prints, suggesting that the morphology of the furrows may depend upon the genetic factor rather than on the environmental influence. This phenomenon corroborates the results of the above investigation in that the natural region had no significant influence on the prints recorded. In the same sense, Moshfeghi et al^13^ have shown the stability of lip wrinkles during a period of 6 months, again demonstrating that despite the environmental changes, the general structure of the lip remains the same.

Regarding the variability of populations, the results of the present study are different from those of Chadha et al,^14^ when comparing Indian and Malay-Chinese subjects, with the result that the distribution of the lips presented significant differences according to sex and race. The results obtained indicate the strong influence of the population and cultural aspect on the morphological characteristics of the grooves, but in our Andean population, no such conditions were obvious. This may be due to the geographic circumstances and the genetic structure of the populations studied. Kumar et al^15^ also noted that the climate of the different regions and their biological adaptations would easily change the density and characteristics of the grooves and therefore the sexual dimorphism in the different human species. On the other hand, recent research has indicated that technological advances facilitate a more precise reading of lip grooves and improve the reproducibility of the results. This finding reinforces the need to adopt standardized protocols and technological tools in the investigation of lip print patterns, especially when comparing classification systems of varying complexity.

Unlike the results of Meena et al^4^ and Thermadam et al,^11^ in which more quadrants revealed relevance, the significant contribution in the present study was made solely by the upper quadrants, confirming the heterogeneity of results in studies in different populations. The conclusions obtained in the present study contribute to the scientific literature since they show that, although sex did not show a significant influence on the patterns of lip prints, the upper quadrants played a significant structural role in the general patterns studied. This should also be considered when forming any future models for the evolution of the influences of several factors that are associated with it.

With respect to classification systems, the data imply that the Suzuki and Tsuchihashi classification may better serve our population in practice, while the Renaud classification is limited in scope despite being more extensive, in that it recognizes only two types of patterns and yields little in the way of significant association. This finding coincides with that of Kapoor et al,^16^ to the effect that the worth of a system of forensic classification lies in its giving rise to results in which there is uniformity and applicability, rather than in its complexity and great number of categories.

Similarly, the analysis of sexual and ecological variables, even though they do not produce significant correlations, gives evidence that the patterns of lip prints appear to behave as an independent character of external influence like the nutrition or the geography, which is important in forensic odontology since it throws some light on the relative to stability of the lip grooves under the contextual conditions, which is the main hypothesis sustained by this paper, and the paper which deals with the association of sex with patterns of lip prints in Quechua and Suni adolescents is not confirmed by the results herewith given, because, while the logistic regression model pointed out the statistical significance of the upper quadrants as contained, in the general pattern, does not show any evidence of the effect of sex on the patterns of lip prints given above. Coupled with this, we must say that cheiloscopy is made to give partial, but not conclusive, proofs of the sexual differentiation in this race, which agrees with what has been noted by Thermadam et al^11^ that this method depends for its applicability on the website of its information or experiment etc. and the numerical size of the sample obtained.

Our results indicated that population/ancestral versus individual differences might be a greater determinant of the sexual differentiation seen in lip prints. One hypothesis to explain the finding that adolescents in the Suni region were less likely to show certain lip print pattern types than adolescents from other regions is that the altitude and ecological conditions of this region have induced either genetic or adaptive differences in the population. Nevertheless, potential confounding variables should be taken into account, such as environmental conditions (cold, dry, etc.), the mixed ethnicity of the populace due to internal migration, and/or the presence of lip pathologies that can change the morphology of the grooves of the lips and, likewise, the classification of the patterns.

This study presents some limitations that must be considered when interpreting the results obtained. The sample size, albeit acceptable, cannot be generalized to other population groups. Second, the study was conducted only in some natural areas of the Ancash Region (Quechua and Suni), which limits the study regarding geographical and environmental representativeness. Regarding the classification systems, we found some limitations in the Suzuki & Tsuchihashi classification system, since it was possible to observe that the concentration in a few patterns diminishes its observable variability. On the contrary, in the case of the system of Renaud, we have more categories, but it was possible to observe that the data were centered in two main types, diminishing the possibility of being able to observe imposing factors. Another of the limitations is the absence of other factors, such as genetic, hormonal or behavioral characteristics that could be influencing the morphology of the lip furrow and that could in turn complete the explanatory power of the model. For these reasons, the results should be understood only as an initial approximation that must be validated with studies of multicentric and larger samples and comparative studies with other classification systems.

The limitations of the current study included not only that the nutrition and weight variables collected weren’t significantly associated with lip print patterns, therefore limiting how much the model on metabolic and body factors could explain, but also that a methodological strength of the current study was acquiring informed consent from parents and assent from adolescents to participate in the study to ensure ethical compliance and that the study process was legitimate. In addition, future studies may be able to overcome the limitations seen in the current study by utilizing advanced imaging methods and digital analysis techniques to achieve more accurate classification and gain insight into how biological variables affect the morphology of lip furrows.

Finally, our study also has limitations due to no significant findings concerning differences between male and female lip prints. This lack of findings limits how well the models explain what they can explain about lip print patterns. It is possible that any differences between males and females (sexual dimorphism) are influenced to a greater extent by population or developmental factors than by sex. Furthermore, the absence of any information regarding the genetic, hormonal or behavioral basis for the development of lip print morphology, limits an understanding of how biology affects lip print form. The limitations of this study are also likely compounded by the fact that advanced digital imaging techniques were not used to assess lip prints, and that further limits the accuracy and repetition of the classification of lip prints. Therefore, future studies should include genetic and hormonal assessments and use standardized digital imaging techniques in order to increase the credibility and interpretability of cheiloscopic evidence.

## Conclusions

In conclusion, the results of the study indicate the existence of specific dominant configurations of lip print patterns in the adolescent population in Ancash, based on what is established by the different classifications used, observing a predominance of types II and IV, in the system of Suzuki & Tsuchihashi and types G and E in the classification of Renaud. There was a significant association between geographic region with respect to lip morphology through logistic regression analysis, indicating that adolescents belonging to the Suni region are less likely to present a general pattern of Suzuki than those belonging to the Quechua region, which suggests a population or ancestral factor that intervenes in the expression of these traits. The rest of the variables studied (sex, age, weight and nutritional status) did not show significant associations, suggesting that the observed differences are attributable to contextual factors rather than individuals. These results contribute to reaffirming the usefulness of the lip prints as a biometric system and also supplying evidence on the morphological diversity of high Andean populations.

## Author contributions

DAT, JM, FM, IC, FMT, FEC, GBY: concept and design of study, drafting, and revision. FEC, GBY, FMT: acquisition of data, analysis, and interpretation. IC, DAT, FMT: acquisition of data, interpretation, and drafting. Finally, all authors had given approval of the version of the article to be published.

## Funding

This research did not receive any specific grant from funding agencies in the public, commercial, or not-for-profit sectors.

## Conflict of interest

The authors declare that they have no known competing financial interests or personal relationships that could have appeared to influence the work reported in this paper.

## References

[1] Jayakrishnan J.M., Reddy J., Vinod Kumar R.B. (2021). Role of forensic odontology and anthropology in the identification of human remains. J Oral Maxillofac Pathol.

[2] Maloth A.K., Dorankula S.P., Pasupula A.P. (2016). Lip outline: a new paradigm in forensic sciences. J Forensic Dent Sci.

[3] Loganadan S., Dardjan M., Murniati N., Oscandar F., Malinda Y., Zakiawati D. (2019). Preliminary research: description of lip print patterns in children and their parents among Deutero-Malay population in Indonesia. Int J Dent.

[4] Meena M., Sharma M., Roy B., Adhapure P.D., Bande Y., Ambulgekar J. (2024). Evaluation of gender determination using lip prints as an adjunctive tool among Indians. Bioinformation.

[5] Mishra P., Panda A., Dash K.C., Kumar H., Bhuyan L., Mahapatra N. (2022). A Cheiloscopy study among students of different regional states in Eastern India: an institutional study. J Pharm Bioallied Sci.

[6] Moore L.G. (2017). Measuring high-altitude adaptation. J Appl Physiol.

[7] Cabezas J.E., Cabezas M., Ureña-López V. (2023). Dermoscopic evaluation of actinic changes in the lips of indigenous children living at high altitude in Ecuador: a descriptive study. Med Sci Monit.

[8] Abedi M., Afoakwah C., Bonsu DNOM (2020). Lip print enhancement: review. Forensic Sci Res.

[9] Ghaferi A.A., Schwartz T.A., Pawlik T.M. (2021). STROBE reporting guidelines for observational studies. JAMA Surg.

[10] Alqarni A.M., Binmahfooz A.M., Alshareef A.A., Alzahrani A.M., Bakhurji E.A. (2024). The reliability of lip print patterns for sex determination: a comparative cross-sectional study. J Forensic Legal Med.

[11] Thermadam A.V., Kumari S., Thomas A.M., Kumar R. (2020). Cheiloscopy in gender determination: a study on 2112 individuals. Egypt J Forensic Sci.

[12] Vanguru R., Pasupuleti S., Manyam R., Supriya A.N., Shrishail B.S., Yoithapprabhunath T.R. (2023). Analysis of inheritance patterns, gender dimorphism and their correlation in lip and palm prints – a cross-sectional study. J Oral Maxillofac Pathol.

[13] Moshfeghi M., Beglou A., Mortazavi H., Bahrololumi N. (2016). Morphological patterns of lip prints in an Iranian population. J Clin Exp Dent.

[14] Chadha R., Kaur J., Garg A. (2022). Lip print evaluation of Indian and Malaysian-Chinese subjects. Egypt J Forensic Sci.

[15] Kumar R., Kaur A. (2023). The role of cheiloscopy in forensic science. J Ophthalmol Eye Care.

[16] Kapoor N., Badiye A. (2017). A study of distribution, sex differences and stability of lip print patterns in an Indian population. Saudi J Biol Sci.

[17] Venkatesh R., David M.P. (2011). Cheiloscopy: an aid for personal identification. J Forensic Dent Sci.

[18] Ata-Ali J., Ata-Ali F. (2014). Forensic dentistry in human identification: a review of the literature. J Clin Exp Dent.

